# Impact of COVID-19 on Physical Activity Among 10,000 Steps Members and Engagement With the Program in Australia: Prospective Study

**DOI:** 10.2196/23946

**Published:** 2021-01-25

**Authors:** Quyen G To, Mitch J Duncan, Anetta Van Itallie, Corneel Vandelanotte

**Affiliations:** 1 Appleton Institute School of Health, Medical and Applied Sciences Central Queensland University Rockhampton Australia; 2 Priority Research Centre for Physical Activity and Nutrition School of Medicine and Public Health The University of Newcastle Newcastle Australia

**Keywords:** exercise, pandemic, lockdown, eHealth, physical activity, COVID-19, engagement, behavior

## Abstract

**Background:**

Physical activity is an important health behavior, due to its association with many physical and mental health conditions. During distressing events, such as the COVID-19 pandemic, there is a concern that physical activity levels may be negatively impacted. However, recent studies have shown inconsistent results. Additionally, there is a lack of studies in Australia on this topic.

**Objective:**

The aim of this study is to investigate changes in physical activity reported through the 10,000 Steps program and changes in engagement with the program during the COVID-19 pandemic.

**Methods:**

Data between January 1, 2018, and June 30, 2020, from registered members of the 10,000 Steps program, which included 3,548,825 days with step data, were used. The number of daily steps were logged manually by the members or synced automatically from their activity trackers connected to the program. Measures on program usage were the number of new registered members per day, the number of newly registered organizations per day, the number of steps logged per day, and the number of step entries per day. Key dates used for comparison were as follows: the first case with symptoms in Wuhan, China; the first case reported in Australia; the implementation of a 14-day ban for noncitizens arriving in Australia from China; the start of the lockdown in Australia; and the relaxing of restrictions by the Australian Government. Wilcoxon signed-rank tests were used to test for significant differences in number of steps between subgroups, between engagement measures in 2019 versus 2020, and before and after an event.

**Results:**

A decrease in steps was observed after the first case in Australia was reported (1.5%; *P*=.02) and after the start of the lockdown (3.4%; *P*<.001). At the time that the relaxing of restrictions started, the steps had already recovered from the lockdown. Additionally, the trends were consistent across genders and age groups. New South Wales, Australian Capital Territory, and Victoria had the greatest step reductions, with decreases of 7.0% (*P*<.001), 6.2% (*P*=.02), and 4.7% (*P*<.001), respectively. During the lockdown, the use of the program increased steeply. On the peak day, there were more than 9000 step entries per day, with nearly 100 million steps logged per day; in addition, more than 450 new users and more than 15 new organizations registered per day, although the numbers decreased quickly when restrictions were relaxed. On average per day, there were about 55 new registered users (*P*<.001), 2 new organizations (*P*<.001), 25.6 million steps (*P*<.001), and 2672 log entries (*P*<.001) more in 2020 compared to the same period in 2019.

**Conclusions:**

The pandemic has had negative effects on steps among Australians across age groups and genders. However, the effect was relatively small, with steps recovering quickly after the lockdown. There was a large increase in program usage during the pandemic, which might help minimize the health impact of the lockdown and confirms the important role of physical activity programs during times of distress and lockdowns.

## Introduction

Physical activity is an important health behavior due to its association with many physical and mental health conditions, such as cardiovascular diseases, cancers, diabetes, depression, and anxiety [1-3]. It is recommended that people should engage in at least 150 minutes of moderate to vigorous physical activity per week [1]. During the COVID-19 pandemic, nearly all aspects of society were impacted. Lockdowns with social isolation measures may result in higher levels of stress and a decline in physical activity [4]. However, it may also mean that time saved from less social gatherings and commuting to work could be used to be more physically active. As physical activity improves mental health [1], it may also be used as a coping mechanism against higher levels of stress and anxiety during the pandemic. In addition, people may engage in physical activity more frequently as it was one of a few accepted reasons for leaving home during the March 2019 lockdown in Australia.

Recent studies have shown inconsistent results regarding changes in physical activity during this pandemic. A report by Fitbit with data from 30 million users worldwide showed a decrease in steps across many countries, including a 4% reduction among Australians [5]. However, the analysis only focused on data during the week of March 22, 2020, and effects of other key events on steps were not reported. In addition, detailed analysis was either not conducted or not reported for Australians. Similarly, a worldwide decrease in mean steps of 5.5% (287 steps) within 10 days and 27.3% (1432 steps) within 30 days of the pandemic declaration was also found among 455,404 smartphone users from 187 countries [6]. Again, very limited information was reported for Australia.

In contrast, one survey among 12,913 participants from 139 countries between March 24 and March 30, 2020, found an increase in frequency of exercising by 88% among those who normally exercise 1 to 2 times per week and 8% among those who normally exercise 3 times per week, but a decrease of 14% among those who normally exercise 4 or more times per week [7]. There were also reports that online searches for topics relating to exercise dramatically increased in Australia, the United Kingdom, and the United States starting on March 23, 2020, when lockdowns started in Australia and the United Kingdom [8]. Data among 50,000 subscribers to the WHOOP platform also showed an increase of 1.1% in exercise frequency and 1.8% in time spent on higher-intensity exercise [9]. Finally, a survey among 1491 Australians found that 49% self-reported a decrease in physical activity, whereas 20.7% reported an increase, during the pandemic, indicating that the impact of COVID-19 may not be consistent among population groups with different demographic characteristics [10].

Given inconsistent findings and lack of detailed studies on this topic in Australia, this study used data from the 10,000 Steps Australia program to investigate (1) changes in physical activity reported through the 10,000 Steps platform during the COVID-19 pandemic and (2) changes in the engagement with the 10,000 Steps program during the COVID-19 pandemic. These findings will improve our understanding of the effect of distressing events on physical activity and engagement in physical activity programs and, therefore, inform health policies and tailored intervention design to better deal with future crises.

## Methods

### Data Sources

The 10,000 Steps program is a web- and mobile-based physical activity promotion program that is funded by the Queensland State Government [11]. To date, the program has registered over 463,000 members (ie, about 3000 new registrations per month), with a total of more than 237 billion steps logged (ie, about 40 million per day). Members register an account with the program website, set a daily step goal, use activity trackers (eg, pedometers, Fitbit, and Garmin) to self-monitor their progress, and participate in challenges to stay motivated. A 10,000 Steps smartphone app is also available for both Apple (iOS) and Google (Android). Any data recorded through the app automatically syncs with the 10,000 Steps website. A detailed description of the program has been published elsewhere [11].

This study used data from new and existing registered members of the program between January 1, 2018, and June 30, 2020, which included 3,548,825 days with step data. Step counts of less than 1000 indicate that the trackers were not worn all day, as even very sedentary people would be expected to accrue more than a 1000 steps a day; in addition, step counts of more than 40,000 are considered as extremely high (eg, technology bug, entry errors, or overreporting) [12,13]. As a result, days with logged steps that were fewer than 1000 steps per day (77,311 days) or more than 40,000 steps per day (10,802 days) were considered invalid and excluded. Data from people not living in Australia (399,711 days), aged less than 18 years or above 80 years (52,551 days), or having less than 7 days of logged data (12,833 days) were also excluded. As a result, 2,995,617 days (84.4%) of data between January 1, 2018, and June 30, 2020, were included in the analysis.

### Measures

Demographic characteristics, including date of birth, gender, and state or territory, were self-reported by the members at the time of registration. Age was calculated by subtracting date of birth from June 30, 2020, and converting to years. Age was then dichotomized into two groups based on the average age: 18-45 years and >45-80 years. Location referred to states and territories in Australia, including, New South Wales, Victoria, Queensland, Northern Territory, Western Australia, South Australia, Tasmania, and Australian Capital Territory.

The number of daily steps was either logged manually by the members (45% through the website and 25% through the app) or synced automatically (30%) from the activity trackers connected to the program. As the 10,000 Steps website and app only extract steps from those using activity trackers (ie, Fitbit and Garmin), and given that many members use mechanical pedometers that only track steps, moderate to vigorous physical activity was not used in the analysis. Other measures used to represent the engagement with the 10,000 Steps program were the number of new registered members per day, the number of newly registered organizations per day, the total number of logged steps per day, and the number of step entries per day [14,15].

Key event dates were selected based on their potential influence on physical activity. The list of COVID-19-related events in Australia is presented in Table 1. The nationwide lockdown was imposed about two months after the first case was reported in Australia at the end of January 2020. During the lockdown, in addition to social distancing guidelines [16], nonessential businesses, such as gyms, indoor sports facilities, and clubs, were closed [17]. People were allowed to be outside only for exercise or other essential needs. Restaurants and cafes only offered takeaway and delivery services.

**Table 1:** Key event dates related to the COVID-19 pandemic.

Date	Description
December 1, 2019	First case with symptoms in Wuhan, China
January 25, 2020	First case in Australia reported
February 5, 2020	A 14-day ban for noncitizens arriving in Australia from China implemented
March 2, 2020	Lockdown starts in Australia
May 8, 2020	Australian Government starts relaxing restrictions

### Data Analyses

Python, version 3.7.6 (Python Software Foundation), was used to process and analyze the data. Mean steps were calculated across users for each day. To smooth out daily fluctuations, a 7-day moving average for daily mean steps was calculated and used to create a time series plot showing the trend in steps over time for the entire sample and for each age group and gender. Key event dates were also marked on the plot. Average steps of 7 and 30 days before and after each event date and percentage of change were calculated and used to indicate the impact of an event on steps. The percentage of change in steps 7 and 30 days before and after the event were reported separately for each age group and gender, as well as for each state and territory. Data on the use of the 10,000 Steps program, including the number of new registered users per day, the number of newly registered organizations per day, the total number of logged steps per day, and the number of step entries per day, were also plotted using 7-day moving averages. Wilcoxon signed-rank tests were used to test for significant differences in steps between subgroups, including men versus women, those 18-45 years of age versus >45-80 years of age, and engagement measures in 2019 versus those in 2020 (ie, the number of new registered users, new registered organizations, total logged steps, and step entries). Significant differences in steps before and after an event as well as before and after the lockdown in each state were also tested using Wilcoxon signed-rank tests. All *P* values were two sided and considered significant if <.05.

## Results

Of 60,560 members logging step data for at least 7 days, 59.7% (n=36,165) were aged between 18 and 45 years and 67.0% (n=40,583) were women. Of 2,995,617 days with step data that were logged between January 1, 2018, and June 30, 2020, about 63.4% (n=1,898,352) were provided by women and 53.1% (n=1,592,021) were provided by those aged between 18 and 45 years.

**Figure 1:**
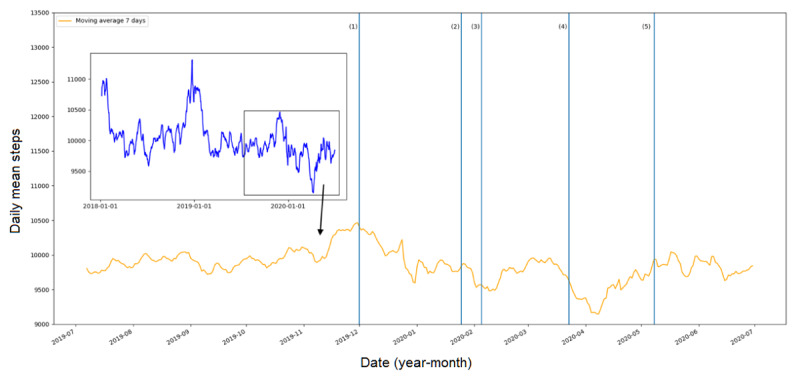
Daily mean steps over time and key COVID-19 pandemic events. (1) First case with symptoms in Wuhan, China; (2) First case in Australia reported; (3) A 14-day ban for noncitizens arriving in Australia from China implemented; (4) Lockdown starts in Australia; (5) Australian Government starts relaxing restrictions.

**Figure 2:**
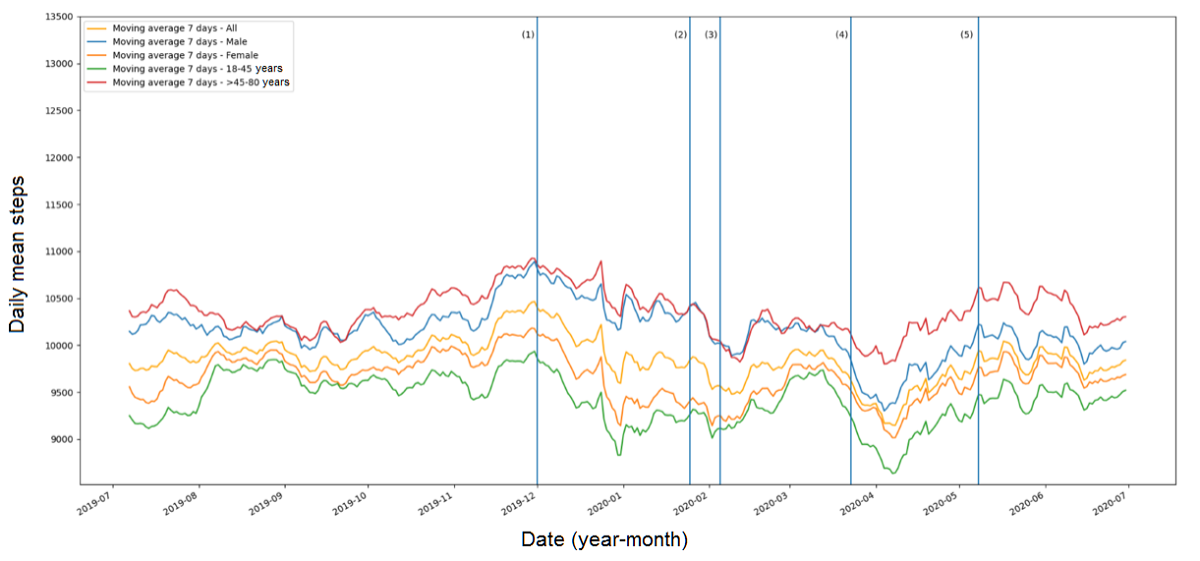
Daily mean steps for subgroups over time and key COVID-19 pandemic events. (1) First case with symptoms in Wuhan, China; (2) First case in Australia reported; (3) A 14-day ban for noncitizens arriving in Australia from China implemented; (4) Lockdown starts in Australia; (5) Australian Government starts relaxing restrictions.

Figure 1 shows the average number of steps per day, zooming in on the period between July 1, 2019, and June 30, 2020. In general, there was a downward trend from the peak at the beginning of December 2019, and the bottom was reached in early April 2020. Since then, an increasing trend was observed. However, the difference in 7-day moving averages between days with the highest and lowest steps was small (ie, <1500 steps). A decrease in steps was also observed after the following events: the first case with symptoms in Wuhan, the first case in Australia reported, and the start of the lockdown in Australia. An increase was observed after a 14-day ban for noncitizens arriving in Australia from China. The steps at the time the Australian Government started relaxing restrictions appeared to have already returned back to the level before the lockdown. With regard to the subgroups, men and those above 45 years of age logged more daily steps than women (difference=505 steps; *P*<.001) and younger adults (difference=930 steps; *P*<.001). However, the trends were consistent for the subgroups and indicated that the effects of COVID-19 were not different for genders and age groups (see Figure 2).

Table 2 shows the average steps of 7 and 30 days before and after an event and for each subgroup. In general, the effects were small for the key events regardless of age group and gender. After the first case was reported in Australia, there was a decrease of 1.5% (*P*=.02) in step average 7 days before and after this date. However, the event with the biggest negative impact on steps was the lockdown starting at the end of March 2020. The reduction was observed for average steps of 7 days (3.4%; *P*<.001) and 30 days (5.0%; *P*<.001) before and after the lockdown. It is also worth noting that the negative impact of a lockdown on steps appeared to be about two times (ie, average of 30 days) larger among those aged between 18 and 45 years compared to older adults. The announcement by the Australian Government to start relaxing restrictions saw a small increase in the step average of 7 days (1.3%; *P*<.001) and 30 days (3.8%; *P*<.001).

**Table 2:** Average daily steps of 7 and 30 days before and after each event.

Event and participants		Step average of 7 days					Step average of 30 days			
		Before, n	After, n	Difference, %	*P* value	Before, n		After, n	Difference, %	*P* value
**First case with symptoms in Wuhan, China**										
	All	10,292	10,193	–1.0	.26	10,238		10,064	–1.7	<.001
	Male	10,707	10,566	–1.3	.22	10,615		10,454	–1.5	.01
	Female	10,011	9937	–0.7	.58	9982		9799	–1.8	<.001
	18-45 years	9737	9591	–1.5	.06	9676		9479	–2.0	<.001
	>45-80 years	10,759	10,701	–0.5	.81	10,718		10,565	–1.4	.004
**First case in Australia reported**										
	All	9778	9634	–1.5	.02	9623		9584	–0.4	.10
	Male	10,377	10,201	–1.7	.09	10,204		10,088	–1.1	.02
	Female	9331	9208	–1.3	.10	9193		9213	0.2	.89
	18-45 years	9219	9117	–1.1	.66	8997		9081	0.9	.25
	>45-80 years	10,358	10,169	–1.8	.003	10,289		10,119	–1.7	<.001
**A 14-day ban for noncitizens arriving in Australia from China implemented**										
	All	9462	9393	–0.7	.02	9452		9465	0.1	.90
	Male	9873	9745	–1.3	.06	9938		9909	–0.3	.41
	Female	9192	9165	–0.3	.16	9134		9175	0.4	.59
	18-45 years	9014	9040	0.3	.80	8955		9057	1.1	.11
	>45-80 years	9950	9780	–1.7	.002	9999		9913	–0.9	.06
**Lockdown starts in Australia**										
	All	9500	9175	–3.4	<.001	9684		9199	–5.0	<.001
	Male	9716	9270	–4.6	<.001	9938		9381	–5.6	<.001
	Female	9391	9130	–2.8	<.001	9555		9112	–4.6	<.001
	18-45 years	9113	8739	–4.1	<.001	9367		8758	–6.5	<.001
	>45-80 years	10,001	9739	–2.6	<.001	10,096		9773	–3.2	<.001
**Australian Government starts relaxing restrictions**										
	All	9637	9767	1.3	<.001	9477		9833	3.8	<.001
	Male	9887	10,035	1.5	.004	9723		10,137	4.3	<.001
	Female	9489	9606	1.2	<.001	9330		9649	3.4	<.001
	18-45 years	9181	9335	1.7	<.001	9060		9426	4.0	<.001
	>45-80 years	10,322	10,415	0.9	.02	10,106		10,446	3.4	<.001

Table 3 shows differences in step averages of 7 days and 30 days before and after the start of the lockdown for each state and territory. A significant decrease was observed in both 7-day and 30-day averages, respectively, for New South Wales (7.0%; *P*<.001 and 5.3%; *P*<.001), Victoria (4.7%; *P*<.001 and 8.1%; *P*<.001), Queensland (1.3%; *P*<.001 and 3.4%; *P*<.001), South Australia (3.2%; *P*=.04 and 2.1%; *P*=.01), Tasmania (3.2%; *P*=.01 and 8.3%; *P*<.001), and Australian Capital Territory (6.2%; *P*=.02 and 10.0%; *P*<.001). The largest decrease for 7 days was seen in New South Wales (about 7%) and the largest decrease for 30 days was seen in Australian Capital Territory (about 10%). No significant difference was found for either Northern Territory (*P*=.15) or Western Australia (*P*=.36).

**Table 3:** Differences in step averages of 7 and 30 days before and after the start of the lockdown for each state.

State	Step average of 7 days				Step average of 30 days				
	Before, n	After, n	Difference, %	*P* value		Before, n	After, n	Difference, %	*P* value
New South Wales	9343	8686	–7.0	<.001		9528	9021	–5.3	<.001
Victoria	9671	9213	–4.7	<.001		9960	9150	–8.1	<.001
Queensland	9216	9098	–1.3	<.001		9399	9084	–3.4	<.001
Northern Territory	9475	8279	–12.6	.15		9744	8969	–8.0	.07
Western Australia	10,519	10,396	–1.2	.36		10,416	10,424	0.1	.69
South Australia	9914	9596	–3.2	.04		10,028	9816	–2.1	.01
Tasmania	9859	9539	–3.2	.01		10,059	9222	–8.3	<.001
Australian Capital Territory	9545	8955	–6.2	.02		9540	8590	–10.0	<.001

**Figure 3:**
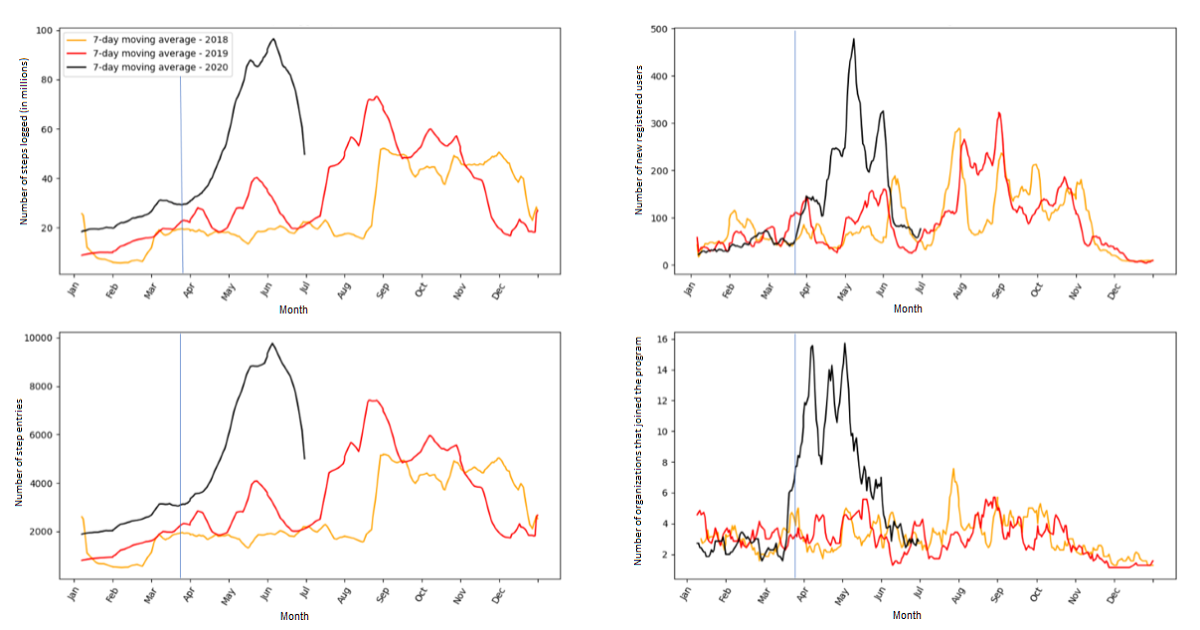
The total number of steps logged, step entries, and new registered users and organizations over time. The vertical line in each graph indicates the start of the lockdown.

On the other hand, the use of the 10,000 Steps program increased during the pandemic (see Figure 3). The number of steps logged per day and the number of step entries increased steeply since the start of the lockdown. At the same time, the number of new registered users and organizations that joined the program also increased sharply. On the peak day, there were more than 9000 step entries with nearly 100 million steps logged per day; in addition, more than 450 new registered users and 15 new organizations registered per day. However, a sharp decrease in the number of logged steps, step entries, and new registered users and organizations was observed since June 2020. On average per day, there were about 55 new registered users (*P*<.001), 2 new registered organizations (*P*<.001), 25.6 million steps (*P*<.001), and 2672 log entries (*P*<.001) more in 2020 compared to the same period in 2019.

## Discussion

### Principal Findings

This study investigated changes in physical activity among the Australian 10,000 Steps members and the use of the program during the COVID-19 pandemic. The results show that, among the key events, the largest decrease in average steps occurred after the lockdown started in Australia. The finding is consistent with the Fitbit report [5] stating that a reduction of 3% to 4% in steps was observed among the participants in Australia during the lockdown. Although the difference between days with the highest and lowest steps was less than 1500 steps, it has been shown that risk reductions in all-cause mortality are between 6% and 36% for every 1000 steps per day increase [18]. However, it is also worth noting that the decrease in steps was only for several months, and the physical activity level among the participants was already high with average daily steps greater than 9000, which are above the national average for Australia [19]. There also seemed to be a decrease in steps even before the lockdown. This may be because the first case of COVID-19 in Australia was reported at the end of January 2020 and the number of cases increased rapidly well before the lockdown in March 2020. This was very prominently reported by the media, so it is likely that many people were already adjusting their behavior before they were imposed to do so by the government. Compared to many other countries that have seen much larger decreases in physical activity [6], the effect during the lockdown was small for Australia. This could be attributed to the fact that Australians were still allowed to exercise outdoors during the pandemic, whereas people from many other countries were not. In addition, at the time of data collection, the Australian Government’s response to COVID-19, ranked third worldwide, was effective not only in keeping infection and death rates low but also in minimizing social disruption compared to many other countries [20]. As a result, people may have felt less worried and were willing to exercise outside. It is also likely that an increase in steps in April 2020 before the end of the lockdown indicated a partial return to normal life as restrictions eased.

The findings also indicate that technology-based (ie, web and app) physical activity promotion programs, such as the 10,000 Steps program, may play a significant role in minimizing the effect of the COVID-19 pandemic on physical activity. As demonstrated in this study, the number of newly registered users reached a record high during the pandemic, along with billions of steps logged monthly. The engagement with the 10,000 Steps program was much higher during the pandemic compared to earlier periods. Consistent with another study reporting an increase in interest in physical activity at the population level [8], our findings support the notion that people were actively searching for alternative ways to stay motivated to engage in physical activity during the pandemic. A possible explanation may be that people were more often choosing to go for a walk as a way to take a break from work or the restrictions of staying at home. It could also be that physical activity was used as a method to cope with stress and anxiety, as physical activity helps reduce stress and improve mental health [1]. A recent study also found that people who self-reported a decrease in physical activity during the pandemic were likely to experience higher stress and depression [10]. As technology-based physical activity programs are able to provide a social platform to support and encourage people to be more active, even when social distancing is mandated, their role is important, especially during distressing times.

In general, the trend in average steps was consistent across age groups and genders during the pandemic. However, the effect of the lockdown appeared to be larger for younger adults. It is likely due to differences in physical activity preferences between the age groups. As older adults are less likely to participate in team-based activities [21] and are more likely to be active at home or go for a walk around their neighborhood compared to using gyms and sporting facilities [22], the effect may be less severe compared to younger adults. In addition, studies have found that older adults were better at emotion regulation and more likely to use positive reappraisal [23,24] (ie, think positively in a negative situation) as a coping strategy [25], which may help them adjust and maintain their lifestyle behaviors, including physical activity in accordance with social isolation measures. It may also be due to the presence of children in younger adults’ households, as studies have shown that parents are likely to be less active compared to nonparents [26,27]. The larger lockdown effect on men could be because men participate more often in competitive physical activities [28], which were more likely to be affected during the pandemic. However, the actual reasons for the observed differences are unclear and more research on this topic is needed.

The immediate effect of the lockdown on step counts was found for most states and territories, with the largest decrease of 7% in New South Wales where a majority of the cases were located in March 2020 [29]. Others with large step count decreases were Victoria, where the second highest number of cases were located, and Australian Capital Territory. Although the number of cases was the third highest in Queensland, the step count decrease was quite small. It is not clear what factors helped, but there was a sharp increase in the use of the 10,000 Steps program, which is based in Queensland. In addition, engagement activities, such as the Queensland Billion Steps Challenge, were implemented by both the government and local organizations. Queensland was also quick in closing the border with New South Wales right after the announcement of the lockdown.

This prospective study included a large number of participants across Australia. Although the main focus was to see the immediate changes in step counts due to COVID-19 events, using several years of data provided additional information on seasonal trends and, therefore, strengthened the results. Time series data also allow examination of both increases and decreases in steps around the key events. However, this study has several limitations. Firstly, many types of physical activity that are dependent on infrastructure that would have closed during the lockdown (eg, swimming pools) were not accounted for by only examining step counts. Secondly, the analysis did not account for differences in lockdown measures between states and territories. Finally, there is a possibility of selection bias, as participation was voluntary. However, the threat of selection bias to validity of this study is minimal due to the fact that our main objective was to see changes in steps over time during the pandemic. When the samples are similar at different time points, the findings will be valid. Furthermore, the sample was large; although a large sample does not mean it is a representative sample, this sample included people from different areas in all states and territories in Australia. Additionally, the 10,000 Steps program supports a variety of step tracking methods and allows both manual entry of steps tracked on any pedometer, activity tracker, or smartphone and automatic syncing of Fitbit and Garmin devices. As such, the risk of selection bias due to the need for an activity tracker or instalment of a specific app was likely low.

### Conclusions

The COVID-19 pandemic had a negative effect on step counts among Australians across age groups and genders. However, the effect was relatively small even during the lockdown; in addition, physical activity levels quickly recovered after the lockdown. There was a significant increase in the use of the 10,000 Steps program during the pandemic, which might help minimize the negative effect of the pandemic and confirm the important role of technology-based physical activity programs during times of distress and lockdowns.
